# The Malarial Question—Letter from Dr. Denman

**Published:** 1897-07

**Authors:** A. M. Denman

**Affiliations:** Lufkin, Texas


### Correspondence.

### The Malarial Question—Letter from Dr. Denman.

LUFKIN, TEXAS, June 14, 1896.

*Editors Texas Medical Journal:*

I have carefully read the paper of Dr. J. E. Gardiner, Medical Sanitary Inspector for Equitable Life Assurance Co., in which he states that he ha ssolved the problem of the mode of infection by malaria; "a misnomer," he calls it, but fails to give it a name. I do not mean to attempt to criticise the paper, but I must say the doctor failed to convince me of the truth of his dogmatic assertion that water was the only way by which the germs could enter the human system. He stated that he resolved to solve the perplexing question by further scientific research and daily observation. I see no scientific research on his part at all; he explodes the idea of science altogether when he says: "Theories, however, are, at best, but logical guesses, and are to the scientist what the exploring shaft is to the miner, or a trail to the huntsman, only a blazed way to the goal. And, as in this instance, possessing the goal—truth, we need no longer the trail or theory." I see no daily observations on his part, as if you will follow him up, he traveled over a territory of 800 miles square, or 64,000 square miles, all in twelve months, eight months of which were not suitable for daily observations of malarial poison. The only statistics which he presents are from saw mill hands who, he says, are leading citizens of the country; and prominent physicians of several places also gave in their evidence; also a merchant who owned a farm on Red River, who wanted it cultivated, gave in his experience; also a banker at Jacksboro handed in what he knew about malarial infection, who, no doubt, knows how to count money and is otherwise intelligent, but knows no more about malaria than I do.

As to Dr. Chas. Smart's article about ice machines preventing malaria, and so on, that is all very good, but the doctor has overlooked the fact that a man has got to be wounded before septicæmia can enter his blood; he has overlooked the truth that an inflamed stomach from impure water and food, with thousands of mineral and vegetable germs in them, does not perform its normal functions; and he should certainly know that if in any machinery the least bolt breaks it stops the machine, but unlike the machine, man does not stop, but goes on working, making other organs do the duty of its crippled member, until finally, from a proper supply of good, pure blood, the lungs, liver, skin, intestines and brain are suitable soil for the reception of malaria. Now we all know that excessive rain will stop the development of malaria, because it arrests decomposition; excessive heat does the same; cold the same, but all things favorable, these germs accumulate rapidly, and in my opinion exist everywhere, in some places in the Southern States so thick that we eat them, breathe them, and even absorb them. Of course this is theory. I do not get this from the farmer. Babies in my county three days old have chills with fevers of malarial types who never drank a drop of water in their short lives—the mother apparently well. It has been said that children in utero have chills while the mother is perfectly well, as far as she knows. In my town we have no wells, no tanks, no creeks or branches, but all have brick cisterns; not a well in town, and we have the old fashioned chills and fevers, pernicious fever, malaria, hematuria, and all the forms of fever due to malaria. I believe, as I said before, that if we boil our water, we would have to drink it boiling, or the germs would get into it so thick while cooling that we would have malaria any way, admitting that water was the only source, or else boil it in bottles and suck it through a quill. I have noticed here for five years that people who eat good, wholesome food in very moderate quantity, and drink small quantities of water; those who do not tank up for breakfast and keep tanked up till dinner on watermelons, peaches, plums and anything they can get, and stay tanked up till dark, not allowing their stomach a moment's rest, are the ones who are immune from not only malaria, but other diseases as well. I believe this germ, malaria, exists anywhere, and will light on any abrasion and enter the blood, and when in sufficient numbers to affect the nervous system and the important organs there will be an effort on nature to expel the offender. High temperature will be the result, and often we see patients who recover from malaria without a dose of medicine.

So, doctor, you see I am a skeptic, and you will have to produce more evidence from a different source before I can accept such dogmatic statements or opinions. I do not care to ask any one to accept my opinion. I would much prefer them to have one of their own as long as they get it by scientific research, not from farmers, bankers and saw-mill hands.

Fraternally yours,

A. M. DENMAN.

